# Optimization of Four Different Rosemary Extraction Techniques Using Plackett–Burman Design and Comparison of Their Antioxidant Compounds

**DOI:** 10.3390/ijms25147708

**Published:** 2024-07-14

**Authors:** Vassilis Athanasiadis, Theodoros Chatzimitakos, Martha Mantiniotou, Dimitrios Kalompatsios, Konstantina Kotsou, Ioannis Makrygiannis, Eleni Bozinou, Stavros I. Lalas

**Affiliations:** Department of Food Science and Nutrition, University of Thessaly, Terma N. Temponera Street, 43100 Karditsa, Greece; vaathanasiadis@uth.gr (V.A.); mmantiniotou@uth.gr (M.M.); dkalompatsios@uth.gr (D.K.); kkotsou@agr.uth.gr (K.K.); ioanmakr1@uth.gr (I.M.); empozinou@uth.gr (E.B.); slalas@uth.gr (S.I.L.)

**Keywords:** *Rosmarinus officinalis*, stirring extraction, ultrasonic bath, ultrasonic probe, pulsed electric field, green extraction techniques, rosmanol, antioxidants, polyphenols, half normal plot

## Abstract

Rosemary has many medicinal and therapeutic properties and therefore it is important to study how to maximize the recovery of its bioactive compounds. In the present study, four different extraction techniques were used, namely stirring extraction (STE), pulsed electric field-assisted extraction (PEF), ultrasound probe-assisted extraction (UPAE), and ultrasound bath-assisted extraction (UBAE). First, some primary experiments were carried out in order to optimize each technique individually through the Plackett–Burman design. Then, each technique was applied under optimal conditions and the results were compared with each other. The optimal total polyphenol content (TPC) of STE is ~19 mg gallic acid equivalents per gram of dry weight (dw), while the antioxidant activity of the extract is 162 μmol ascorbic acid equivalents (AAEs) per gram of dw via FRAP and ~110 μmol AAE per gram of dw via DPPH. As for PEF, the optimal TPC is ~12 mg GAE/g dw, and the FRAP and DPPH values are ~102 and ~70 μmol AAE per gram of dw, respectively. When it comes to UPAE, the optimal TPC is ~16 mg GAE/g dw and the antioxidant capacity of the extract is ~128 μmol AAE/g dw through FRAP and ~98 μmol AAE/g dw through DPPH. UBAE optimal extract yielded ~17 mg GAE/g dw TPC, ~146 μmol AAE/g dw for FRAP, and ~143 μmol AAE/g dw for DPPH. The highest flavonoid content (~6.5 mg rutin equivalent/g dw) and DPPH (~143 μmol ascorbic acid equivalent/g dw) is obtained through UBAE. UPAE has been shown to be more efficient in recovering ascorbic acid (~20 mg/g dw). Additionally, the chlorophyll-to-carotenoid ratios of UPAE and UBAE were 2.98 and 2.96, respectively, indicating that the extracts had a generally positive impact on health. Considering the environmental impact of each extraction technique but also which antioxidant factor needs to be maximized, the most suitable extraction technique will be chosen.

## 1. Introduction

Herbal medicines and natural products were employed in ancient therapies [1]. *Rosmarinus officinalis* (RO) L. or rosemary is a member of the Lamiaceae family and is widely distributed [2]. It has been demonstrated to possess neuroprotective, antidepressant, and stress-relieving properties, as well as provide relief from headache, stomachache, memory loss, and physical and mental fatigue [3,4,5]. The documented medicinal properties of rosemary, either in animal models or in cultured cells, validate its bioactivity [5]. In recent decades, researchers have focused more on herbs in drug discovery due to their limited side effects and fewer complications [6], which has led to a great deal of interest in RO and its potential for further consideration and utilization. In addition, RO extracts have significant value in the food sector. In 2008, rosemary extracts were authorized by the European Food Safety Authority (EFSA) of the European Union as a food additive in several categories of food products, bearing the code name E392 [7]. Furthermore, the Joint FAO/WHO Expert Committee on Food Additives (JECFA), in 2016, assessed E392 and established a provisional daily intake (ADI) of 0–0.3 mg/kg of body weight (BW) [7].

It is well established that the biological properties of RO are primarily due to phenolic compounds it contains [8]. RO leaves have been extensively studied for their bioactive compounds, including total polyphenols and total flavonoids, as well as their ascorbic acid content [9,10,11]. Kasparavičienė et al. [9] found that a simple stirring process yielded a 50% ethanol extract of RO leaves with a total polyphenol content of 49 ± 1.05 mg rosmarinic acid equivalent/mL. In a later study, Nadia and Rachid [10] developed an RO leaf extract with simple stirring too, and a solvent of 30% ethanol: 70% water. They quantified the total flavonoids, finding a value of 9.075 ± 0.002 mg quercetin equivalent/g. Finally, most recently, Soltanabad et al. [11] extracted RO leaves of different seasons using the same method with ethanol as the solvent. Their findings revealed that ascorbic acid can range from 0.09 mg/g in winter to 0.11 mg/g in summer.

In recent years, green extraction techniques have gained significant importance, especially in the food, pharmaceutical, and cosmetic industries, due to the rising demand for naturally derived products that are produced sustainably and in an environmentally friendly manner [12,13]. These techniques encompass sustainable methods for extracting valuable components from natural sources like plants, herbs, and fruits [14]. Compared to traditional extraction methods, green extraction techniques can result in higher yields of target compounds and generate less waste [15,16,17]. Furthermore, by using green innovative extraction methods, lower amounts of solvents are consumed and less extraction time is required [14,18]. Common green extraction methods include ultrasonic extraction, both ultrasonic probe-assisted extraction [19] and ultrasonic bath-assisted extraction [20]; pulsed electric field [21]; microwave-assisted extraction [22]; enzyme-assisted extraction [22]; hydrostatic pressure extraction [22]; pressurized liquid extraction [23]; and supercritical fluid-assisted extraction [22].

In light of the nutritional value of RO, its potential beneficial properties, and its possible important applications in the food and pharmaceutical industries, further analysis is necessary to optimize its extraction in order to enhance the maximum value of various nutrients and bioactive elements. Furthermore, given the value of studying and exploiting green extraction techniques, the primary objective of this research is to examine four different extraction techniques, including conventional stirring, ultrasonic probe-assisted extraction, ultrasonic bath-assisted extraction, and pulsed electric field, for the extraction of RO leaves. Plackett–Burman design was implemented to simultaneously evaluate the significance of multiple variables and investigate their impact on RO leaf extraction. Instead of developing a comprehensive single-factor model, screening studies usually employ two levels of characterization to identify important variables [24]. Since it enables the assessment of interactions between variables, this design is ideal for investigating two-level multiple variables. Additionally, this approach decreases data collection by eliminating any less significant components [25]. By optimizing the techniques and all extraction parameters, like the suitable solvent, the solvent-to-solid ratio, the extraction duration, and the number of extraction cycles, along with the individual parameters of each technique, a fully nutrient-enhanced RO leaf extract will be obtained, which will be an excellent natural additive for food and pharmaceutical products.

## 2. Results and Discussion

### 2.1. Extraction Techniques

In the present study, the extraction of bioactive compounds from rosemary leaves was carried out using four different techniques, one conventional (stirring extraction) and three green (pulsed electric field, ultrasonication probe, and ultrasonication bath-assisted extraction) techniques. Initially, twelve preliminary experiments through the Plackett–Burman design were conducted in order to optimize the parameters of each technique. All extracts were analyzed for their total polyphenol content (TPC) and antioxidant capacity by ferric-reducing antioxidant power (FRAP) and 1,1-diphenyl-2-picrylhydrazyl scavenging (DPPH^•^) assays. Afterwards, the four techniques were applied again, under the optimal conditions. Optimizing the duration and temperature of extraction is essential for reducing the energy expenditure involved in the process. The consensus is that higher temperatures have a beneficial effect on extraction processes by increasing the solubility of solutes and improving diffusion coefficients. It is crucial to acknowledge that there is a limit at which phenolic compounds can experience degradation [26]. A thorough analysis is necessary to clarify the effects of time on individual extraction. In previous research, it is stated that both short and extended extraction durations are effective [27]. However, it is crucial to investigate whether the repetition of the extraction process would increase the recovery of bioactive compounds or not.

#### 2.1.1. Stirring Extraction (STE) Technique

**Figure 1:**
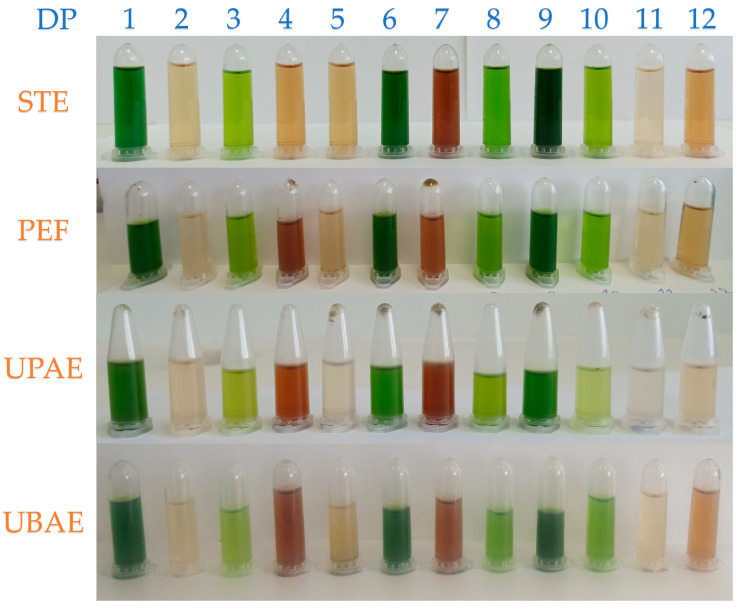
Sample extracts were obtained using four (4) different extraction techniques and twelve (12) design points (DPs) from the Plackett–Burman design. STE—stirring extraction; PEF—pulsed electric field-assisted extraction; UPAE—ultrasonic probe-assisted extraction; UBAE—ultrasonic bath-assisted extraction.

The first extraction technique under investigation is a classic one, stirring extraction (STE) [28]. The parameters under examination are the solvent composition (A1), which is either water or ethanol, the solvent-to-solid ratio (A2), the extraction time (A3), the particle size of the rosemary sample (A4), the temperature of the extraction (A5), the stirring speed (A6), the size of the magnetic bar (A7), and the number of the extraction cycles (A8). The extraction solvent and the solvent-to-solid ratio are two of the most studied parameters when it comes to extraction optimization, as they both influence the amount of bioactive compounds recovered [26]. Another very important factor affecting the extraction process is the number of extraction cycles. Therefore, increasing the number of extraction cycles could increase the recovery of bioactive compounds, but considering the cost and environmental impact of this process, it is imperative to investigate the need to repeat extractions to increase efficiency [29]. Figure 1 shows the extracts that were taken from each distinct design point using each extraction technique. Table 1 shows the results of the TPC, FRAP, and DPPH^•^ for every design point, regarding only STE. The TPC recoveries range from 3.95 to 23.42 mg GAE/g dw, while FRAP varies from 22.54 to 202.47 μmol AAE/g dw, and DPPH^•^ from 11.86 to 112.75 μmol AAE/g dw. It is observed that the higher TPC and FRAP values are obtained from design point 7, but for DPPH^•^, it is obtained from design point 5. The differences between the two design points lie in variables A2, A3, A4, and A6. The solvent-to-solid ratio, particle size, extraction duration, and stirring speed are likely to be key parameters that affect extraction. The duration and temperature of the extraction process, as well as the solvent-to-solid ratio, have been proven to significantly impact the amount of extracted bioactive compounds [30].

**Figure 2:**
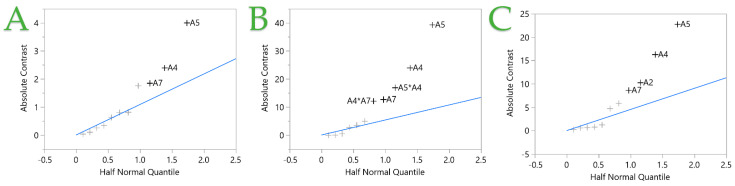
Half normal plot of the stirring extraction (STE) technique from fit two-level screening report. Plots (**A**–**C**) represent the TPC, FRAP, and DPPH assays, respectively. Independent variables (+) with nonzero means and places outside the blue line are considered significant.

The half normal plot in the fit two-level screening platform plots the absolute values of contrasts against the absolute values of the quantiles for the half-normal distribution. The blue line passes through the origin with a slope equal to Lenth’s estimate of σ (standard deviation). Small effects are referred to as error terms and are presumptively distributed normally, with a mean of zero and a standard deviation of σ. The blue line corresponds to these terms. Effects that do not lie on the blue line and have nonzero means are considered significant. In Figure 2, the factors that significantly affect the STE are depicted. It is obvious that factors A4, A5, and A7 strongly affect the extraction of polyphenols and antioxidants, while in the FRAP assay, the combination of factors A4*A7 and A5*A4 also affect the antioxidant recovery. Furthermore, in Figure 2C, it can be observed that factor A2 also influences the recovery of antioxidant compounds. 

#### 2.1.2. Pulsed Electric Field (PEF)-Assisted Extraction Technique

Recent research has extensively examined sustainable approaches to utilize food and food waste, which can effectively reduce pollution and harness the potential of bioactive substances for numerous applications in the food business, pharmaceuticals, and other fields [31]. The implementation of non-thermal extraction techniques, specifically pulsed electric field (PEF)-assisted extraction, presents a hopeful and eco-friendly approach to retrieve valuable bioactive substances from food. This paves the way for a more effective and sustainable future in food processing [32,33]. In this context, PEF is also one of the green techniques examined in this study. Some of the examined PEF parameters are the same as STE (A1–A4 and A8, B1–B4 and B8), where additional studies targeting PEF parameters include the electric field strength (B5), the pulse period (B6), and the pulse duration (B7). PEF is particularly suitable for the recovery of thermolabile substances due to its operation at a moderate electric field, such as 0.5 and 1 kV/cm [34]. However, there is an inverse relationship between field strength and pulse width; lower field strength with wider pulses can produce similar results to higher field strength with narrower pulses [35]. Thus, it is imperative to optimize the conditions under which the PEF-assisted extraction will take place, to maximize the recovery of bioactive compounds.

**Figure 3:**
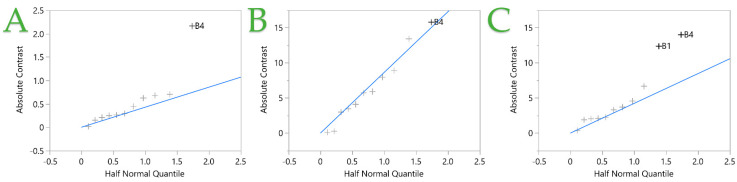
Half normal plot of the pulsed electric field (PEF)-assisted extraction technique from fit two-level screening report. Plots (**A**–**C**) represent the TPC, FRAP, and DPPH assays, respectively. Independent variables (+) with nonzero means and places outside the blue line are considered significant.

Table 2 presents the TPC, FRAP, and DPPH^•^ results obtained from each design point, while Figure 3 shows the half normal plot applied to these results. The TPC recoveries range from 5.78 to 12.26 mg GAE/g dw, while FRAP varies from 21.87 to 112.55 μmol AAE/g dw and DPPH^•^ from 16.10 to 99.10 μmol AAE/g dw. In Table 2, it is evident that the maximum polyphenol recovery is on design point 10, while the maximum antioxidant recovery from both FRAP and DPPH^•^ is obtained on design point 1. In Figure 3, it is highlighted that the only factor that has a significant impact on all three recoveries is the medium particle size (B4). As observed in STE and in PEF, the smaller particle size leads to higher extraction yields. The DPPH^•^ assay is also affected by the composition of the solvent, and more specifically, ethanol seems to give higher DPPH^•^ yields.

#### 2.1.3. Ultrasonic Probe-Assisted Extraction (UPAE) Technique

Ultrasound-assisted technology is regarded as a straightforward technique with reduced extraction time and increased yield. The probe system has a higher intensity of energy across a smaller surface area, specifically the tip of the ultrasound probe. Thus, it has the capability to minimize energy dissipation, hence enhancing the efficacy of the ultrasonic treatment in the extraction process [36]. Ultrasound probes can vary in terms of tip geometries, probe diameter, and length. The choice of probes is determined by the specific characteristics and quantity of the sample employed in the ultrasound sonication procedure [37]. The UPAE parameters studied were the same as those of PEF and STE (X1-X4 and X8), except that the additional parameters studied were the ultrasonic power (C5), pulsation (C6), and the probe length position (C7).

**Figure 4:**
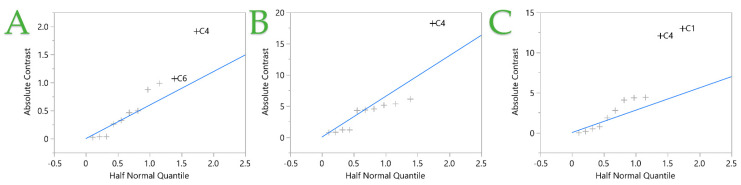
Half normal plot of the ultrasonic probe-assisted extraction (UPAE) technique from fit two-level screening report. Plots (**A**–**C**) represent the TPC, FRAP, and DPPH assays, respectively. Independent variables (+) with nonzero means and places outside the blue line are considered significant.

Table 3 provides the yields of TPC, FRAP, and DPPH^•^ of the experiments. The TPC recoveries range from 3.35 to 12.90 mg GAE/g dw, while FRAP varies from 20.26 to 100.78 μmol AAE/g dw and DPPH^•^ from 11.83 to 83.42 μmol AAE/g dw. It is observed that the higher TPC and FRAP yields are obtained through design point 4, but for the DPPH^•^, design points 1 and 3 seem to be more favorable. Figure 4 shows that once again, the medium particle size of rosemary leaves plays a crucial role to the recovery of bioactive compounds. The TPC recovery is also affected by the pulsation, while the antioxidant scavenging activity is affected by the solvent, as observed in the PEF technique. In general, when ethanol is applied as a solvent, enhanced antiradical scavenging activity is obtained.

#### 2.1.4. Ultrasonic Bath-Assisted Extraction (UBAE) Technique

Ultrasounds, like other sound waves, disperse as a sequence of compression and rarefaction waves that spread through the molecules of the material they are exposed to [38]. When the intensity is strong, the cycles of rarefaction overpower the attraction interactions between the molecules in the medium, resulting in the formation of cavitation bubbles. When the collapse occurs, cavitation bubbles generate fast-moving jets that lead to the destruction of cellular structures and enable the penetration of solvents [39]. The physical parameters of the solvent, including viscosity, surface tension, and saturation vapor pressure, have an impact on cavitation [38]. Both ultrasonic bath-assisted extraction (UBAE) and UPAE are subject to the same principle. Nevertheless, the ultrasonic probe system does have certain drawbacks. For instance, immersing the probe directly in the sample will result in a quicker temperature increase throughout the extraction process, as there is less energy lost to the surrounding environment compared to using an ultrasonic bath system [36,37]. Hence, it is important to also study UBAE in order to clarify whether different extraction yields ultimately arise, and if so, which of the two techniques is more efficient. The examined parameters targeting UBAE are ultrasonic power (D5), ultrasonic frequency (D6), and ultrasonic mode (D7).

**Figure 5:**
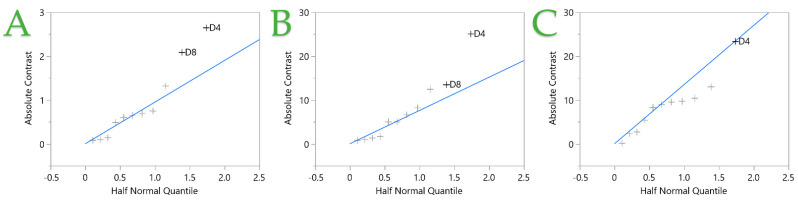
Half normal plot of the ultrasonic bath-assisted extraction (UPAE) technique from fit two-level screening report. Plots (**A**–**C**) represent the TPC, FRAP, and DPPH assays, respectively. Independent variables (+) with nonzero means and places outside the blue line are considered significant.

Table 4 represents the extraction yields of UBAE. The TPC recoveries range from 6.16 to 18.71 mg GAE/g dw, while FRAP varies from 46.59 to 169.40 μmol AAE/g dw and DPPH^•^ from 21.82 to 139.46 μmol AAE/g dw. Design point 5 has proved to be the most efficient for TPC, FRAP, and DPPH^•^ yields. In Figure 5, it is shown that other than medium particle size, which affects all yields, the other parameter that affects the TPC and FRAP yields is the repetition of the extraction.

### 2.2. Partial Least Squares (PLS) Analysis

#### 2.2.1. Prediction Profiler

**Figure 6:**
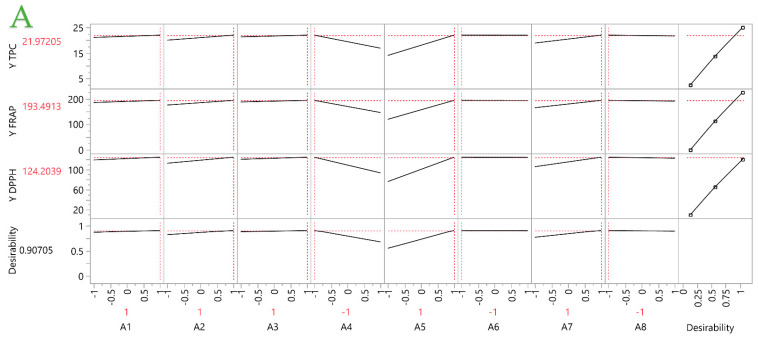

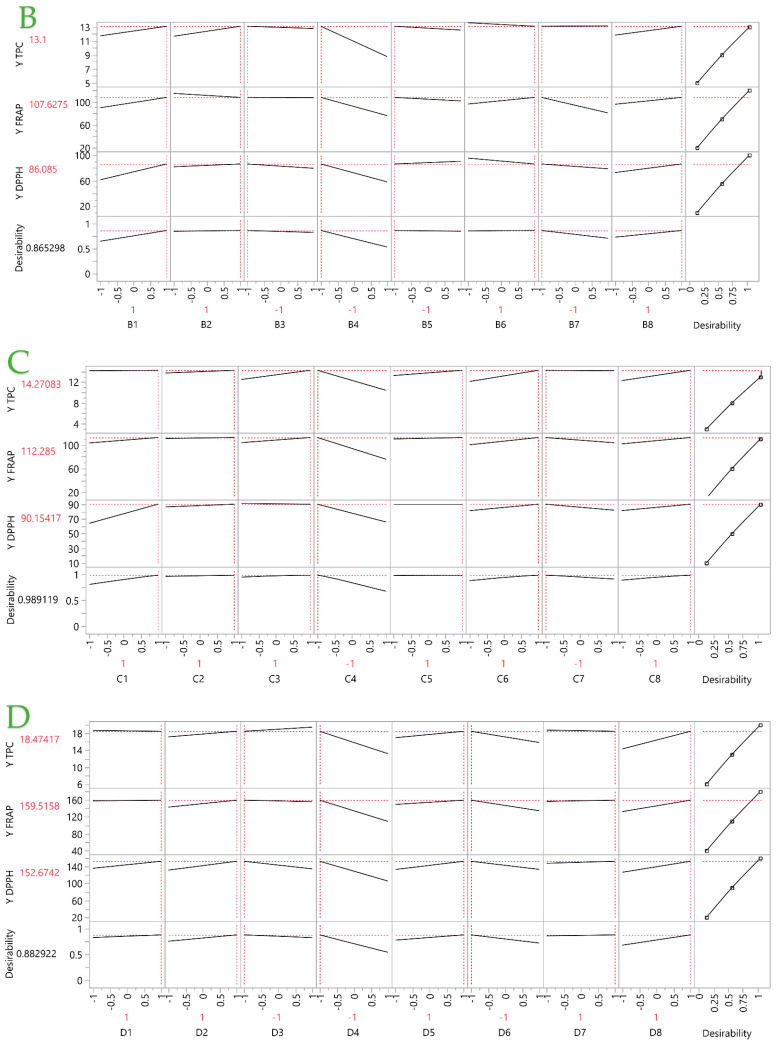
Prediction profiler for four different extraction techniques using partial least squares (PLS) analysis. The techniques of STE, PEF, UPAE, and UBAE are represented, in corresponding plots, by (**A**–**D**). The slopes of the lines for each predictor reflect the model coefficients.

**Figure 7:**
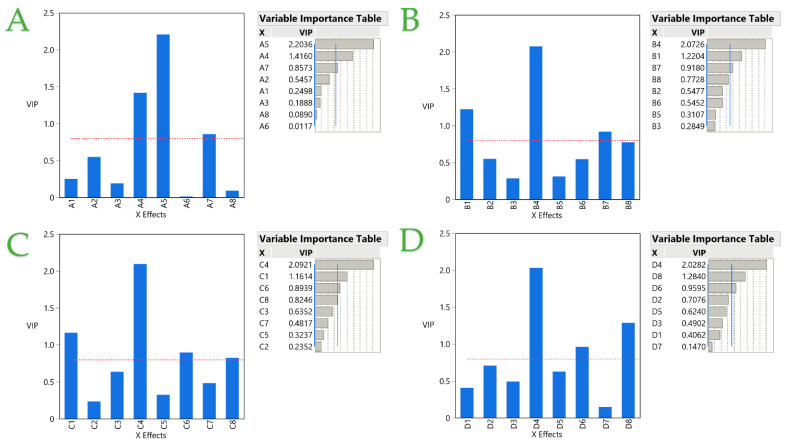
The variable importance plot (VIP) values for every predictor variable as well as the VIP scores are shown in the VIP option graph within the VIT. The significance level for each variable is shown by a red dashed line in each plot or a blue line in each VIT at 0.8. The techniques of STE, PEF, UPAE, and UBAE are represented, in corresponding plots, by (**A**–**D**).

A partial least squares (PLS) analysis was employed to the obtained results to achieve optimization of each extraction technique. Regarding STE (Figure 6A), the optimal conditions are formed as ethanol as the solvent, 50 mL/g solvent-to-solid ratio, 120 min extraction time, 0.4 mm particle size, at 80 °C and 500 rpm, 25 mm magnet size, and only one extraction cycle. This model poses a great fit, with a desirability of ~0.91. The PEF optimal conditions (Figure 6B) are ethanol as the solvent, 50 mL/g, 10 min, and two extraction cycles, utilizing 0.4 mm powder, applying 0.6 kV/cm, and with a pulse period of 1000 μs and pulse duration of 10 μs. The desirability is ~0.87, which implies a good fit for the model. In Figure 6C, the optimal conditions of UPAE are presented. These are ethanol as the solvent, 50 mL/g, two extraction cycles of 20 min each, 0.4 mm particle size, operating at 120 W with pulsation 48 pulses/min and 5 mm probe length position from the bottom. This model provides an excellent fit to the model, as its desirability is ~0.99. Moreover, the optimal UBAE conditions (Figure 6D) are formed as ethanol solvent, two extraction cycles 5 min each, 0.4 mm average particle size at 50 mL/g solvent-to-solid ratio, operating at 220 W with 37 kHz frequency at pulse mode. The desirability of this model is ~0.88, implying a good fit for the model. Figure 7 depicts a variable importance plot for each technique, and it is once again denoted that the particle size of rosemary is the one factor that impacts the extraction at all four different techniques. Furthermore, it is evident that the solvent applied is important when it comes to PEF and UPAE, while the number of extraction cycles only enhances the UBAE. Extraction temperature has a significant effect only in the case of STE, where higher temperatures lead to higher extraction yields.

#### 2.2.2. Analysis of the Optimal Extracts

After optimization of each individual technique, the four extractions were carried out, under the optimal conditions, and their results were analyzed. In Table 5, the predicted PLS optimal values are provided, along with the parameters that significantly affect the extractions. In Table 6, the optimal results of the extractions are provided. The TPC acquired by STE is 18.57 ± 1.21 mg GAE/g dw, which closely matches the value of the PLS predictor. Calderón-Oliver et al. [16] assessed a TPC which was ~27.3% lower than ours, when a conventional extraction technique was employed, utilizing 95% ethanol as a solvent. It is noteworthy that this is the highest performance among all the approaches, along with the FRAP value. Nevertheless, the DPPH value did not reach the maximum value perceived. Furthermore, the optimal STE extract had the largest concentration of chlorophylls. This is also supported by the color measurements, which showed that this extract had the most vibrant green color (Table 6). In nearly all of the analyses conducted, PEF exhibited the lowest recoveries, rendering it the least effective of the four techniques. Despite having a greater chlorophyll concentration compared to UBAE, the chlorophyll a/b ratio of the PEF optimal extract is approximately 41% lower. Additionally, it was noticed that the PEF optimal extract exhibited the dimmest green color. Regarding UPAE, its TPC is around 9.9% greater than expected by the PLS model, its FRAP is about 18.6% higher, and its DPPH is approximately 8.7% higher. The UPAE technique demonstrated the highest recovery of ascorbic acid, with UBAE and STE techniques following closely after. In addition, UPAE yielded the highest chlorophyll a/b ratio, as well as the second-highest total chlorophyll and carotenoid content. The ultrasonic color measurements of both procedures yielded comparable results, with UPAE exhibiting a little deeper shade. More significantly, the extracts with the highest total carotenoid content were UPAE and UBAE. These two techniques also showed the lowest chlorophyll-to-carotenoid ratios. The balance between these substances in plant extracts may improve their general health-promoting qualities, which makes the ratio of chlorophylls to carotenoids important [40]. As for UBAE, it also led to results that were highly similar to the ones anticipated by the PLS model. It has the second-greatest TPC recovery, which is not significantly different from the highest recovery achieved by STE. Furthermore, UBAE exhibited the most substantial recovery of total flavonoids from rosemary. Additionally, it is worth noting that the ascorbic acid content (AAC) did not show any significant differences compared to STE and UPAE. The two ultrasonic procedures yielded higher total carotenoid content values compared to the other two techniques, with UPAE once again surpassing UBAE by ~17%. Jacotet-Navarro et al. [41] also assessed the effects of ultrasound-probe and ultrasound-bath systems on the extraction of certain phenolic acids from rosemary, under the same conditions. The results indicate that ultrasound probe is more efficient than ultrasound bath, and this may be attributed to the high power/intensity generation of probe-based systems.

The identification and quantification of individual polyphenols and flavonoids by HPLC-DAD is also of interest. The results are shown in Table 7. Initially, it is shown that the compound with the highest content in all extracts is rosmanol, ranging from 3.47 to 3.79 mg/g, followed by rosmarinic acid (2.85–3.11 mg/g) and hesperidin (1.26–2.24 mg/g). Also, it becomes evident that STE did not favor the extraction of epicatechin and quercetin 3-*D*-galactoside. This can most likely be attributed to the elevated temperature applied during extraction, which probably may have caused the degradation of these compounds, as polyphenols and flavonoids are known to be susceptible to elevated temperatures [42]. The greatest rosmanol quantity was recovered by UPAE and STE, but there was no statistically significant difference (*p* > 0.05) among the rosmanol quantities of all extraction techniques. However, there seems to be a statistically significant difference (*p* > 0.05) in the rosmarinic acid quantity, where the highest values were observed for UPAE and UBAE (3.11 and 3.04, respectively), but are not far from STE and PEF (2.85 and 2.92, respectively). Hesperidin was mostly favored by STE, followed by UPAE and UBAE, and lastly by PEF. Moreno et al. [43] implemented steam-distillation extraction with water as a solvent, and the residues were subjected to Soxhlet extraction using acetone, and they quantified 0.079 mg rosmarinic acid/g dw through HPLC. This result, compared to the four techniques examined in this study, highlights not only the high performance of these techniques, but also their environmental sustainability. Based on the findings of this study, it appears that overall, all four techniques examined provide comparable results, and the selection of the most appropriate one depends on which factor needs to be maximized. If the intent is to maximize polyphenol yield, then STE is the optimal option, as it results in the highest feasible yield. However, when taking into account environmental considerations or other factors like AAC or antiradical activity, PEF, UPAE, and UBAE appear to be more appropriate alternatives. Among the above options, UPAE delivers higher results compared to the other two.

### 2.3. Principal Component Analysis (PCA)

**Figure 8:**
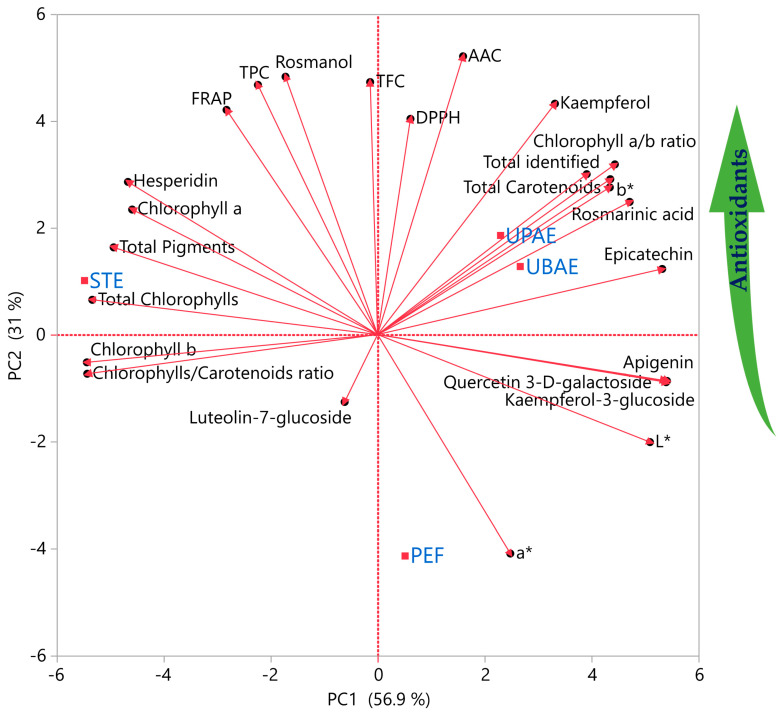
Principal component analysis (PCA) for the measured parameters for four different extraction techniques (with blue color).

To conduct an extensive analysis of the data and extract additional information, PCA was employed, the results of which are depicted in Figure 8. The primary objective was to identify whether a correlation between the variables under investigation (i.e., bioactive compounds, antioxidant activity, and extraction techniques) was observed. The graph explained the 87.9% of the variance. It was observed that several polyphenols were positioned far from each other and in close proximity with extraction techniques, indicating that different polyphenols were abundant with extraction techniques. For instance, rosmarinic acid, epicatechin, and kaempferol were mostly extracted under both ultrasonication techniques, whereas hesperidin and pigments were extracted with conventional stirring. In addition, both ultrasonication techniques had positive correlation with total carotenoids, indicating a higher extraction yield of these compounds compared to other extraction techniques. It should also be noted that the discrimination of the different extraction techniques was a matter of antioxidant activity, revealing that UPAE and UBAE were the most successful techniques in yielding antioxidant compounds. Similar results were obtained in the study from Karabegović et al. [44], in which the bioactive compounds extractive yield from cherry laurel leaf and fruit was higher than conventional extraction.

### 2.4. Multivariate Correlation Analysis (MCA)

**Figure 9:**
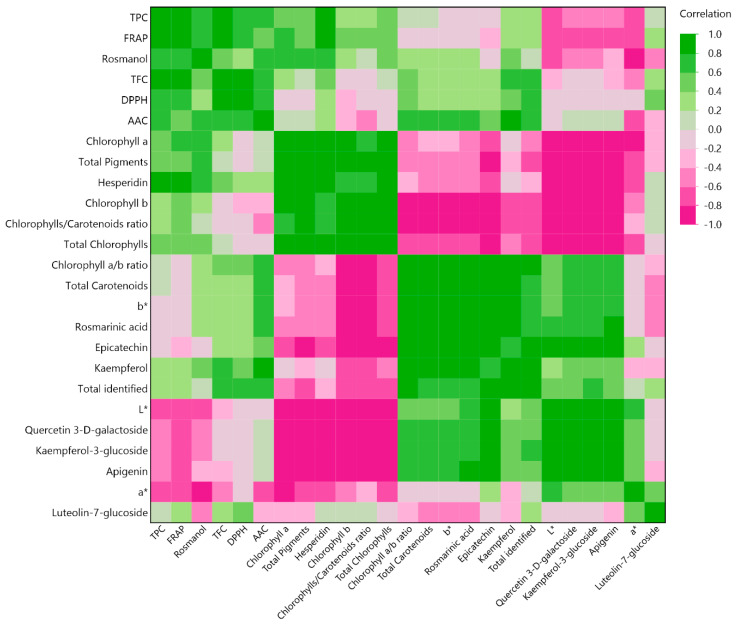
Multivariate correlation analysis of measured parameters.

To provide further insight to the correlation between the variables, the MCA diagram was employed. Its capacity to measure the extent of positive or negative correlation among the variables is a major advantage of this approach. The color scale from pink to green indicates correlation values from −1 to 1, respectively, the results of which are illustrated in Figure 9. Total chlorophylls and hesperidin were observed to have a strong negative correlation (>0.8) with individual polyphenols, such as quercetin-3-*D*-galactoside, kaempferol-3-glucoside, and apigenin, indicating that these compounds were affected by different extraction parameters (i.e., technique or solvent). An interesting finding was that DPPH^•^ scavenging activity did not show any strong correlation with any bioactive compound, including individual polyphenols and ascorbic acid. Regarding the color of the extracts, a highly expected trend was revealed, as it was shown that the ascending concentration of chlorophylls and other pigments negatively impacted the lightness of the extracts. However, it was also revealed with a lower correlation (>0.6) that the darker the extract, the higher the TPC value.

## 3. Materials and Methods

### 3.1. Chemicals and Reagents

Methanol, hydrochloric acid, trichloroacetic acid, L-ascorbic acid, aluminum chloride, 2,4,6-tris(2-pyridyl)-*s*-triazine (TPTZ), 2,2-diphenyl-1-picrylhydrazyl (DPPH^•^), and all chemical standards for the HPLC determination of polyphenols were obtained from Sigma-Aldrich (Darmstadt, Germany). Gallic acid, ethanol, and the Folin–Ciocalteu reagent were bought from Panreac Co. (Barcelona, Spain). Acetonitrile was obtained from Labkem (Barcelona, Spain). From Merck (Darmstadt, Germany), iron (III) chloride was purchased. Formic acid (98%) and anhydrous sodium carbonate were purchased from Penta (Prague, Czech Republic). Deionized water from a deionizing column was used for all conducted experiments.

### 3.2. Instrumentation

A Biobase BK-FD10P freeze-dryer (Jinan, China) was used to lyophilize RO leaves. The sieving process was performed using a vibratory sieve shaker Fritsch Analysette 3 (Fritsch GmbH, Idar-Oberstein, Germany). Conventional stirring was performed by a hotplate (Heidolph Instruments GmbH & Co. KG, Schwabach, Germany) for the STE. Two custom stainless-steel chambers (Val-Electronic, Athens, Greece), a mode/arbitrary waveform generator (UPG100, ELV Elektronik AG, Leer, Germany), a digital oscilloscope (Rigol DS1052E, Beaverton, OR, USA), and a high-voltage power generator were used to perform the PEF extraction. A Biobase UCD-150 ultrasonic cell disrupter (Jinan, China) with a maximum nominal power of 150 W, equipped with a probe tip (emitting surface) diameter of 6 mm was used to conduct all extractions for the UPAE. An Elmasonic P70H ultrasonic bath (Elma Schmidbauer GmbH, Singen, Germany) was used to conduct all extractions for the UBAE. A Shimadzu UV-1900i double-beam UV-Vis spectrophotometer (Kyoto, Japan) was used for all spectrophotometric analyses. A Shimadzu CBM-20A liquid chromatograph and a Shimadzu SPD-M20A diode array detector, both provided by Shimadzu Europa GmbH in Duisburg, Germany, were utilized for the quantification of individual polyphenols. A Phenomenex Luna C18(2) column (Torrance, California, USA) maintained at 40 °C, was used to separate the compounds chromatographically (100 Å, 5 μm, and 4.6 mm × 250 mm). A colorimeter (Lovibond CAM-System 500, the Tintometer Ltd., Amesbury, UK), was used for the determination of CIELAB parameters (L*, a*, and b*) from the RO extracts.

### 3.3. Collection and Handling of RO Leaves

RO leaves were obtained from a local plant shop from Karditsa region (Central Greece). After rinsing the leaves with distilled water, they were subsequently dried with paper towels and finally freeze-dried. The fresh RO leaves were found to have a moisture content of 78.62 ± 1.18%. The leaves were ground into a fine powder, sieved, and separated into two fractions with medium average sizes of 0.4 mm and 1.2 mm, respectively. Lastly, the powder was kept at a temperature of –40 °C until additional analysis was conducted.

### 3.4. RO Leaves Extraction Procedure through Plackett–Burman Design

The optimal conditions from each extraction technique were identified to extract the majority of the bioactive compounds from RO leaves. Eight extraction parameters were studied to determine their impact on the extraction of bioactive compounds, as shown in Table 8. The parameters were represented by two values, with 1 indicating the maximum and –1 indicating the minimum. The extraction procedures shared several common characteristics, including X1, X2, X3, X4, and X8. These factors were associated with the extraction solvent, the ratio of solvent-to-solid, the extraction time, the particle size, and the number of extraction cycles. The parameters X5, X6, and X7 are related to specific conditions of each extraction process.

### 3.5. Bioactive Compounds Determination

#### 3.5.1. Total Polyphenol Content (TPC)

Total polyphenol content (TPC) was expressed as mg gallic acid equivalents (GAE) per gram of dry weight (dw), based on a previous study [26]. Briefly, 100 μL of Folin–Ciocalteu reagent was mixed with 100 μL of properly diluted sample, and after exactly 2 min, 800 μL of 5% *w*/*v* aqueous sodium carbonate solution was added in a 2 mL Eppendorf tube. The absorbance was measured spectrophotometrically at 740 nm after the mixture was incubated at 40 °C for 20 min. Utilizing a gallic acid calibration curve (10–100 mg/L in methanol, y = 0.0138x − 0.0044, R^2^ = 0.9996), the total polyphenol concentration (*C*_TP_) was determined. The following Equation (1) was employed to determine TPC as mg gallic acid equivalents (GAE) per gram of dry weight (dw):

(1) $$\mathrm{TPC}\;(\mathrm{mg}\;\mathrm{GAE}/g\;\mathrm{dw})=\frac{C_{TP}\;\times \;V}{w}$$

where the volume of the extraction medium is denoted as *V* (in L) and the dry weight of the sample as *w* (in grams).

#### 3.5.2. Total Flavonoid Content (TFC)

Total flavonoid content (TFC) was determined based on a previously established technique [45] and was calculated as mg rutin equivalents (RtE) per gram of dry weight (dw). In brief, a volume of 100 μL of the properly diluted sample was mixed with 860 μL of aqueous ethanol (35% *v*/*v*) and 40 μL of a reagent that included 5% (*w*/*v*) aluminum chloride and 0.5 M sodium acetate. The mixture was left at ambient temperature for 30 min before measuring the absorbance at 415 nm. A rutin (quercetin 3-*O*-rutinoside) calibration curve (30–300 mg/L in methanol, y = 0.003x + 0.0053, R^2^ = 0.9966) was used to measure total flavonoid concentration (*C*_TFn_). The TFC was expressed as mg rutin equivalents (RtE) per gram dry weight (dw), using the following Εquation (2):

(2) $$\mathrm{TFC}\;(\mathrm{mg}\;\mathrm{RtE}/g\;\mathrm{dw})=\frac{C_{\mathrm{TFn}}\;\times \;V}{w}$$

where *V* is the volume of the extraction medium (in L) and *w* is the dry weight of the sample (in grams).

#### 3.5.3. HPLC Quantification of Polyphenolic Compounds

High-performance liquid chromatography (HPLC) of individual polyphenols from the RO extracts was based on our prior research [26]. The mobile phase consisted of 0.5% formic acid in acetonitrile (B) and 0.5% formic acid in aqueous solution (A). The gradient program involved gradual initiation from 0 and increase to 40% B, followed by 50% B in 10 min, 70% B in another 10 min, and a constant value for 10 min. The mobile phase flow rate was kept constant at 1 mL/min. By comparing the absorbance spectrum and retention time to those of purified standards, the compounds were identified and subsequently quantified using calibration curves (0–50 μg/mL). Satisfactory R^2^ values (>0.99) were observed for all identified compounds.

#### 3.5.4. Ascorbic Acid Content (AAC)

Ascorbic acid content from samples was quantified as mg/g of dried weight, as previous with described by Athanasiadis et al. [27]. A quantity of 500 μL of 10% (*v*/*v*) Folin–Ciocalteu reagent along with 100 μL of properly diluted sample extract were mixed with 900 μL of 10% (*w*/*v*) trichloroacetic acid in an Eppendorf tube. The absorbance was immediately measured at 760 nm after 10 min of storage in the absence of light. The calibration curve of ascorbic acid had a linear range of 50–500 mg/L, linear equation y = 0.0016 − 0.0085, and R^2^ 0.9980.

#### 3.5.5. Total Pigment Concentration

Total chlorophylls and carotenoids were extracted from ~1 g of RO powder with ethanol as the solvent. Properly diluted samples underwent a spectrophotometric full scan from 200 to 800 nm. Based on the recalculated specific absorption coefficient for ethanol as a solvent, as reported in the Wellburn and Lichtenthaler (1984) study [46], the following equations are derived:

(3) $$C_{a}\;(\mu g/\mathrm{mL})=(13.36\;\times \;A_{664}\;\times \;F_{D})\;-\;(5.19\;\times \;A_{649}\;\times \;F_{D}),\;C_{a}\left(\mu g/g\mathrm{dw}\right)=\frac{C_{a}\;\times \;V}{w}$$

(4) $$C_{b}\;(\mu g/\mathrm{mL})=(27.43\;\times \;A_{649}\;\times \;F_{D})\;-\;(8.12\;\times \;A_{664}\;\times \;F_{D}),\;C_{b}\left(\mu g/g\mathrm{dw}\right)=\frac{C_{b}\;\times \;V}{w}$$

(5) $$C_{a+b}\;(\mu g/\mathrm{mL})=(5.24\;\times \;A_{664}\;\times \;F_{D})\;+\;(22.24\;\times \;A_{649}\;\times \;F_{D}),\;C_{a+b}\left(\mu g/g\mathrm{dw}\right)=\frac{C_{a+b}\;\times \;V}{w}$$

(6) $$C_{x+c}\;(\mu g/\mathrm{mL})=\frac{(1000\;\times \;A_{470}\times \;F_{D})-(2.13\;\times \;C_{a})-(97.64\;\times \;C_{b})}{209},\;C_{x+c}\left(\mu g/g\mathrm{dw}\right)=\frac{C_{x+c}\;\times \;V}{w}$$

where *C*_a_ is chlorophyll a concentration; *C*_b_ is chlorophyll b concentration; *C*_a+b_ is the total chlorophylls concentration; *C*_x+c_ is the total carotenoids (xanthophylls + β-carotene) concentration; *A* is the absorbance in a quartz cell of 1 cm; *F*_D_ is the dilution factor of extracts; *V* is the volume of the extraction medium (in mL); and *w* is the weight of the dry weight (in grams).

#### 3.5.6. Color Analysis of the Extracts

The color measurement of the extracts was conducted using a previous methodology [45]. A colorimeter was used to measure the CIELAB parameters (L*, a*, and b*) for the RO extracts. Three parameters are used to describe color: The perceived lightness of a color is denoted by the L* value, which ranges from 0 (representing absolute black) to 100 (representing absolute white). The a* value quantifies the extent to which a color is green (positive values) or red (negative values). In a similar way, the b* value quantifies how much a color leans toward blue (positive values) or yellow (negative values).

### 3.6. Antioxidant Activity of the Extracts

#### 3.6.1. Ferric-Reducing Antioxidant Power (FRAP) Assay

The ferric-reducing antioxidant power (FRAP) was calculated as μmol of ascorbic acid equivalents (AAE) per gram of dw based on a previously established methodology by Shehata et al. [47]. Briefly, 50 μL of FeCl_3_ solution (4 mM in 0.05 M HCl) was combined with 50 μL of the appropriately diluted sample in a 1.5 mL Eppendorf tube. After incubating the mixture at 37 °C for 30 min, 900 μL of TPTZ solution (1 mM in 0.05 M HCl) was promptly added. The absorbance was measured at 620 nm after 5 min. The ferric-reducing power (*P*_R_) was determined by employing an ascorbic acid calibration curve (*C*_AA_) in 0.05 M HCl (50–500 μM, y = 0.0019x − 0.0005, R^2^ = 0.9997). Equation (7) was employed to evaluate the *P*_R_ in μmol of ascorbic acid equivalents (AAE) per gram of dw:

(7) $$P_{R}\;(\mu \mathrm{mol}\;\mathrm{AAE}/g\;\mathrm{dw})=\frac{C_{AA}\;\times \;V}{w}$$

where *V* is denoted (in L) as the volume of the extraction medium and *w* represents the weight of the dried material (in grams).

#### 3.6.2. DPPH^•^ Antiradical Activity Assay

The antiradical activity from bioactive compounds for DPPH^•^ scavenging (expressed as μmol AAE per gram of dw) was evaluated based on a previous procedure [47]. In brief, 1950 μL of a 100 μM DPPH^•^ solution in methanol were mixed with 50 μL of the sample, with the solution being stored at room temperature for 30 min in the absence of light. The absorbance was measured at 515 nm. In addition, the use of a blank sample was appropriate (consisting of DPPH^•^ solution and methanol), with the absorbance being immediately being measured. For the calculation of the scavenging percentage, Equation (8) was employed:

(8) $$\%\;\mathrm{Scavenging}=\frac{A_{\mathrm{control}}-A_{\mathrm{sample}}}{A_{\mathrm{control}}}\times 100$$

Antiradical activity (*A*_AR_) was evaluated through an ascorbic acid calibration curve (100–1000 μM of ascorbic acid, y = 0.0576x + 0.7960, R^2^ = 0.9926). Ascorbic acid concentration (*C*_AA_) was expressed as μmol AAE per gram of dw:

(9) $$A_{\mathrm{AR}}\;(\mu \mathrm{mol}\;\mathrm{AAE}/g\;\mathrm{dw})=\frac{C_{AA}\;\times \;V}{w}$$

where *V* is denoted (in L) as the volume of the extraction medium and *w* represents the weight of the dried material (in grams).

### 3.7. Statistical Analysis

The Plackett–Burman design statistical analysis was carried out using JMP^®^ Pro 16 software (SAS, Cary, NC, USA). The extraction processes were carried out at least twice for every batch and extraction technique of RO extracts, and the quantitative analysis was carried out three times. The Kolmogorov–Smirnov test was used to determine whether the data were normal. Using the Tukey HSD multiple comparison test, a one-way analysis of variance (ANOVA) was carried out to ascertain whether there were any significant differences. Averages and measures of variability are used to present the results. With the JMP^®^ Pro 16 software, statistical analyses of the half normal plot, partial least squares (PLS) analysis, principal component analysis (PCA), and multivariate correlation analysis (MCA) were carried out.

## 4. Conclusions

The overall results of this study indicate that four distinct extraction techniques, three environmentally friendly and one traditional, result in yields that are somewhat comparable. The standard technique, known as STE, demonstrates certain strengths, but their distinctions are quite minor. When taking into account the expenditure of energy and time, it may be more advantageous to choose for a more environmentally friendly technique that may achieve comparable yields while minimizing energy waste. Among the various environmentally friendly techniques, ultrasound-based approaches, particularly UPAE, appear to be advantageous for extracting multiple antioxidant compounds, including polyphenols, flavonoids, carotenoids, and ascorbic acid all simultaneously. Although there is a plethora of research dealing with the extraction of bioactive and antioxidant compounds utilizing green techniques, the part of the most sustainable technique for each plant is still missing. Furthermore, the implementation of ethanol seems to facilitate the extraction of chlorophylls, while also favoring the antiradical activity of the extracts. Additional organic solvents, such as hexane, acetone, and methanol, can be utilized independently or in combination with water for the purpose of extraction. The selection of solvents should be based on critical factors, such as the specific component to be extracted (e.g., hexane would be the preferred choice if tocopherols are targeted), while also considering the potential harm they may have to both humans and the environment. The results of our study demonstrate the considerable potential of rosemary as a valuable source of bioactive and antioxidant compounds in the food and pharmaceutical industries.

## Figures and Tables

**Table 1:** Experimental findings for the eight independent variables under investigation and the dependent variable’s responses to the stirring extraction (STE) technique.

Design Point	Independent Variables ^1^								Responses ^2^		
	A1	A2	A3	A4	A5	A6	A7	A8	TPC	FRAP	DPPH
1	100 (1)	10 (−1)	30 (−1)	0.4 (−1)	80 (1)	250 (−1)	10 (−1)	2 (1)	14.98	132.85	90.07
2	0 (−1)	50 (1)	30 (−1)	1.2 (1)	80 (1)	500 (1)	10 (−1)	1 (−1)	10.55	94.22	59.22
3	100 (1)	50 (1)	30 (−1)	0.4 (−1)	20 (−1)	500 (1)	10 (−1)	1 (−1)	9.76	95.81	66.54
4	0 (−1)	10 (−1)	120 (1)	1.2 (−1)	20 (−1)	500 (1)	10 (−1)	2 (1)	4.90	34.09	25.89
5	0 (−1)	50 (1)	30 (−1)	1.2 (−1)	80 (1)	250 (−1)	25 (1)	2 (1)	19.63	188.05	112.75
6	100 (1)	10 (−1)	30 (−1)	0.4 (1)	20 (−1)	500 (1)	25 (1)	2 (1)	7.95	66.19	30.31
7	0 (−1)	10 (−1)	120 (1)	0.4 (−1)	80 (1)	500 (1)	25 (1)	1 (−1)	23.42	202.47	103.57
8	100 (1)	50 (1)	120 (1)	1.2 (1)	80 (1)	500 (1)	25 (1)	2 (1)	14.46	106.49	94.47
9	100 (1)	10 (−1)	120 (1)	1.2 (1)	80 (1)	250 (−1)	10 (−1)	1 (−1)	12.70	111.08	54.92
10	100 (1)	50 (1)	120 (1)	0.4 (−1)	20 (−1)	250 (−1)	25 (1)	1 (−1)	13.44	89.91	77.40
11	0 (−1)	50 (1)	120 (1)	1.2 (1)	20 (−1)	250 (−1)	10 (−1)	2 (1)	7.71	54.67	29.79
12	0 (−1)	10 (−1)	30 (−1)	1.2 (1)	20 (−1)	250 (−1)	25 (1)	1 (−1)	3.95	22.54	11.86

^1^ A1: ethanol concentration in %, *v*/*v*; A2: solvent-to-solid ratio in mL/g; A3: extraction time in min; A4: medium particle size in mm; A5: temperature in °C; A6: stirring speed in rpm; A7: magnetic stir bar in mm; A8: number of cycles. ^2^ Total polyphenol content (TPC) in mg GAE/g dw; ferric reducing antioxidant power (FRAP) in μmol AAE/g dw; 2,2-Diphenyl-1-picrylhydrazyl (DPPH) in μmol AAE/g dw.

**Table 2:** Experimental findings for the eight independent variables under investigation and the dependent variable’s responses to the pulsed electric field (PEF)-assisted extraction technique.

Design Point	Independent Variables ^1^								Responses ^2^		
	B1	B2	B3	B4	B5	B6	B7	B8	TPC	FRAP	DPPH
1	100 (1)	10 (−1)	10 (−1)	0.4 (−1)	1 (1)	100 (−1)	10 (−1)	2 (1)	12.02	112.55	99.10
2	0 (−1)	50 (1)	10 (−1)	1.2 (1)	1 (1)	1000 (1)	10 (−1)	1 (−1)	5.78	44.70	28.35
3	100 (1)	50 (1)	10 (−1)	0.4 (−1)	0.6 (−1)	1000 (1)	10 (−1)	1 (−1)	11.66	91.14	68.30
4	0 (−1)	10 (−1)	30 (1)	1.2 (−1)	0.6 (−1)	1000 (1)	10 (−1)	2 (1)	10.66	92.69	49.56
5	0 (−1)	50 (1)	10 (−1)	1.2 (−1)	1 (1)	100 (−1)	100 (1)	2 (1)	11.38	29.76	63.20
6	100 (1)	10 (−1)	10 (−1)	0.4 (1)	0.6 (−1)	1000 (1)	100 (1)	2 (1)	6.53	48.94	47.30
7	0 (−1)	10 (−1)	30 (1)	0.4 (−1)	1 (1)	1000 (1)	100 (1)	1 (−1)	7.57	55.23	34.17
8	100 (1)	50 (1)	30 (1)	1.2 (1)	1 (1)	1000 (1)	100 (1)	2 (1)	8.82	49.79	46.89
9	100 (1)	10 (−1)	30 (1)	1.2 (1)	1 (1)	100 (−1)	10 (−1)	1 (−1)	5.36	37.78	42.99
10	100 (1)	50 (1)	30 (1)	0.4 (−1)	0.6 (−1)	100 (−1)	100 (1)	1 (−1)	12.26	61.52	72.04
11	0 (−1)	50 (1)	30 (1)	1.2 (1)	0.6 (−1)	100 (−1)	10 (−1)	2 (1)	6.92	49.88	36.34
12	0 (−1)	10 (−1)	10 (−1)	1.2 (1)	0.6 (−1)	100 (−1)	100 (1)	1 (−1)	6.14	21.87	16.10

^1^ B1: ethanol concentration in %, *v*/*v*; B2: solvent-to-solid ratio in mL/g; B3: extraction time in min; B4: medium particle size in mm; B5: electric field strength in kV/cm; B6: pulse period in μs; B7: pulse duration in μs; B8: number of cycles. ^2^ Total polyphenol content (TPC) in mg GAE/g dw; ferric reducing antioxidant power (FRAP) in μmol AAE/g dw; 2,2-Diphenyl-1-picrylhydrazyl (DPPH) in μmol AAE/g dw.

**Table 3:** Experimental findings for the eight independent variables under investigation and the dependent variable’s responses to the ultrasonic probe-assisted extraction (UPAE) technique.

Design Point	Independent Variables ^1^								Responses ^2^		
	C1	C2	C3	C4	C5	C6	C7	C8	TPC	FRAP	DPPH
1	100 (1)	10 (−1)	5 (−1)	0.4 (−1)	120 (1)	12 (−1)	5 (−1)	2 (1)	10.67	95.17	83.42
2	0 (−1)	50 (1)	5 (−1)	1.2 (1)	120 (1)	48 (1)	5 (−1)	1 (−1)	7.19	56.19	34.08
3	100 (1)	50 (1)	5 (−1)	0.4 (−1)	60 (−1)	48 (1)	5 (−1)	1 (−1)	8.96	81.59	80.54
4	0 (−1)	10 (−1)	20 (1)	1.2 (−1)	60 (−1)	48 (1)	5 (−1)	2 (1)	12.90	100.78	57.86
5	0 (−1)	50 (1)	5 (−1)	1.2 (−1)	120 (1)	12 (−1)	15 (1)	2 (1)	9.38	68.12	43.29
6	100 (1)	10 (−1)	5 (−1)	0.4 (1)	60 (−1)	48 (1)	15 (1)	2 (1)	6.58	56.23	54.71
7	0 (−1)	10 (−1)	20 (1)	0.4 (−1)	120 (1)	48 (1)	15 (1)	1 (−1)	11.37	80.37	46.04
8	100 (1)	50 (1)	20 (1)	1.2 (1)	120 (1)	48 (1)	15 (1)	2 (1)	10.84	65.01	58.15
9	100 (1)	10 (−1)	20 (1)	1.2 (1)	120 (1)	12 (−1)	5 (−1)	1 (−1)	4.95	45.82	39.80
10	100 (1)	50 (1)	20 (1)	0.4 (−1)	60 (−1)	12 (−1)	15 (1)	1 (−1)	9.63	87.01	66.30
11	0 (−1)	50 (1)	20 (1)	1.2 (1)	60 (−1)	12 (−1)	5 (−1)	2 (1)	6.97	50.29	33.65
12	0 (−1)	10 (−1)	5 (−1)	1.2 (1)	60 (−1)	12 (−1)	15 (1)	1 (−1)	3.35	20.26	11.83

^1^ C1: ethanol concentration in %, *v*/*v*; C2: solvent-to-solid ratio in mL/g; C3: extraction time in min; C4: medium particle size in mm; C5: ultrasonic power in W; C6: pulsation (ON/OFF) in pulses/min; C7: probe length position in mm; C8: number of cycles. ^2^ Total polyphenol content (TPC) in mg GAE/g dw; ferric reducing antioxidant power (FRAP) in μmol AAE/g dw; 2,2-Diphenyl-1-picrylhydrazyl (DPPH) in μmol AAE/g dw.

**Table 4:** Experimental findings for the eight independent variables under investigation and the dependent variable’s responses to the ultrasonic bath-assisted extraction (UBAE) technique.

Design Point	Independent Variables ^1^								Responses ^2^		
	D1	D2	D3	D4	D5	D6	D7	D8	TPC	FRAP	DPPH
1	100 (1)	10 (−1)	5 (−1)	0.4 (−1)	220 (1)	37 (−1)	1 (−1)	2 (1)	17.42	128.76	123.58
2	0 (−1)	50 (1)	5 (−1)	1.2 (1)	220 (1)	80 (1)	1 (−1)	1 (−1)	8.24	57.44	46.11
3	100 (1)	50 (1)	5 (−1)	0.4 (−1)	110 (−1)	80 (1)	1 (−1)	1 (−1)	9.02	89.96	76.28
4	0 (−1)	10 (−1)	20 (1)	1.2 (−1)	110 (−1)	80 (1)	1 (−1)	2 (1)	15.69	108.86	63.12
5	0 (−1)	50 (1)	5 (−1)	1.2 (−1)	220 (1)	37 (−1)	2 (1)	2 (1)	18.71	169.40	139.46
6	100 (1)	10 (−1)	5 (−1)	0.4 (1)	110 (−1)	80 (1)	2 (1)	2 (1)	7.91	65.08	47.17
7	0 (−1)	10 (−1)	20 (1)	0.4 (−1)	220 (1)	80 (1)	2 (1)	1 (−1)	10.30	77.11	42.39
8	100 (1)	50 (1)	20 (1)	1.2 (1)	220 (1)	80 (1)	2 (1)	2 (1)	11.29	73.50	67.82
9	100 (1)	10 (−1)	20 (1)	1.2 (1)	220 (1)	37 (−1)	1 (−1)	1 (−1)	8.99	71.00	39.60
10	100 (1)	50 (1)	20 (1)	0.4 (−1)	110 (−1)	37 (−1)	2 (1)	1 (−1)	15.17	123.43	95.96
11	0 (−1)	50 (1)	20 (1)	1.2 (1)	110 (−1)	37 (−1)	1 (−1)	2 (1)	11.93	82.52	37.54
12	0 (−1)	10 (−1)	5 (−1)	1.2 (1)	110 (−1)	37 (−1)	2 (1)	1 (−1)	6.16	46.59	21.82

^1^ D1: ethanol concentration in %, *v*/*v*; D2: solvent-to-solid ratio in mL/g; D3: extraction time in min; D4: medium particle size in mm; D5: ultrasonic power in W; D6: ultrasonic frequency in kHz; D7: ultrasonic mode, 1 for sweep and 2 for pulse; D8: number of cycles. ^2^ Total polyphenol content (TPC) in mg GAE/g dw; ferric reducing antioxidant power (FRAP) in μmol AAE/g dw; 2,2-Diphenyl-1-picrylhydrazyl (DPPH) in μmol AAE/g dw.

**Table 5:** Maximum desirability for every variable under each optimal extraction condition for four different extraction techniques, as determined by the partial least squares (PLS) prediction profiler.

Technique	Independent Variables ^1^								PLS Model Values ^2^		
	X1	X2	X3	X4	X5	X6	X7	X8	TPC	FRAP	DPPH
STE	1	1	1	−1	1	−1	1	−1	21.97	193.49	124.20
PEF	1	1	−1	−1	−1	1	−1	1	13.10	107.63	86.09
UPAE	1	1	1	−1	1	1	−1	1	14.27	112.29	90.15
UBAE	1	1	−1	−1	1	−1	1	1	18.47	159.52	152.67

^1^ The significance variables for each technique from the variable importance plot (VIP) analysis are indicated by values in red; ^2^ Total polyphenol content (TPC) in mg GAE/g dw; ferric reducing antioxidant power (FRAP) in μmol AAE/g dw; 2,2-Diphenyl-1-picrylhydrazyl (DPPH) in μmol AAE/g dw.

**Table 6:** Different parameters under each optimal extraction condition for four different extraction techniques.

Parameters	STE	PEF	UPAE	UBAE
TPC (mg GAE/g dw)	18.57 ± 1.21 ^a^	11.94 ± 0.82 ^c^	15.68 ± 1.08 ^b^	17.34 ± 1.20 ^a,b^
TFC (mg RtE/g dw)	5.62 ± 0.15 ^b^	3.21 ± 0.24 ^d^	5.01 ± 0.12 ^c^	6.46 ± 0.14 ^a^
FRAP (μmol AAE/g dw)	161.95 ± 3.56 ^a^	101.65 ± 3.05 ^d^	127.69 ± 6.51 ^c^	146.36 ± 7.46 ^b^
DPPH (μmol AAE/g dw)	110.1 ± 6.61 ^b^	69.86 ± 3.63 ^c^	97.96 ± 3.33 ^b^	142.65 ± 5.71 ^a^
AAC (mg/g dw)	18.32 ± 1.15 ^a,b^	16.23 ± 0.81 ^b^	20.01 ± 0.86 ^a^	19.3 ± 0.68 ^a^
Chlorophyll a (μg/g dw)	849.31 ± 56.05 ^a^	608.98 ± 17.66 ^c^	713.13 ± 33.52 ^b^	595.46 ± 18.46 ^c^
Chlorophyll b (μg/g dw)	744.64 ± 45.42 ^a^	500.31 ± 12.51 ^b^	396.97 ± 10.32 ^c^	347.09 ± 15.97 ^c^
Chlorophyll a/b ratio	1.14 ± 0.01 ^d^	1.22 ± 0.00 ^c^	1.8 ± 0.04 ^a^	1.72 ± 0.03 ^b^
Total chlorophylls (μg/g dw)	1593.95 ± 66.95 ^a^	1109.29 ± 43.26 ^b^	1110.1 ± 62.17 ^b^	942.55 ± 33.93 ^c^
Total carotenoids (μg/g dw)	215.39 ± 7.32 ^c^	240.2 ± 18.01 ^c^	372.36 ± 23.83 ^a^	317.89 ± 21.3 ^b^
Chlorophylls/carotenoids ratio	7.4 ± 0.06 ^a^	4.62 ± 0.17 ^b^	2.98 ± 0.02 ^c^	2.96 ± 0.09 ^c^
Total Pigments (μg/g dw)	1809.34 ± 74.27 ^a^	1349.49 ± 61.28 ^b,c^	1482.46 ± 86 ^b^	1260.44 ± 55.23 ^c^
L*	57 ± 0.6 ^c^	73 ± 0.4 ^a^	70.7 ± 0.8 ^b^	71.2 ± 0.6 ^b^
a*	−19.7 ± 0.5 ^c^	−13.2 ± 0.5 ^a^	−19.5 ± 0.5 ^c^	−14.9 ± 0.4 ^b^
b*	41.9 ± 0.8 ^c^	43 ± 0.5 ^c^	47.9 ± 0.4 ^a^	45.6 ± 0.8 ^b^
Color ^1^				

Statistically significant figures (*p* < 0.05) are indicated with lowercase letters (e.g., a–d) within each row. ^1^ The L*, a*, and b* measured values were used to fill the table cells with the extract’s matching color using the appropriate HEX code.

**Table 7:** Polyphenolic compounds (mg/g dw) under each optimal extraction condition for four different extraction techniques.

Polyphenolic Compound	STE	PEF	UPAE	UBAE
Epicatechin	n.d. *	0.05 ± 0.00 ^b^	0.08 ± 0.00 ^a^	0.09 ± 0.01 ^a^
Quercetin 3-*D*-galactoside	n.d.	1.03 ± 0.07 ^a^	1.13 ± 0.08 ^a^	1.11 ± 0.03 ^a^
Luteolin-7-glucoside	0.57 ± 0.03 ^b^	0.58 ± 0.03 ^b^	0.07 ± 0.00 ^c^	0.85 ± 0.02 ^a^
Kaempferol-3-glucoside	1.12 ± 0.07 ^a^	1.24 ± 0.08 ^a^	1.25 ± 0.09 ^a^	1.26 ± 0.07 ^a^
Hesperidin	2.24 ± 0.10 ^a^	1.26 ± 0.04 ^c^	1.54 ± 0.04 ^b^	1.51 ± 0.11 ^b^
Rosmarinic acid	2.85 ± 0.08 ^b^	2.92 ± 0.11 ^a,b^	3.11 ± 0.07 ^a^	3.04 ± 0.06 ^a,b^
Apigenin	0.76 ± 0.03 ^a^	0.82 ± 0.04 ^a^	0.83 ± 0.03 ^a^	0.82 ± 0.04 ^a^
Kaempferol	0.97 ± 0.03 ^a^	0.95 ± 0.05 ^a^	1.01 ± 0.04 ^a^	1.00 ± 0.04 ^a^
Rosmanol	3.79 ± 0.27 ^a^	3.47 ± 0.11 ^a^	3.80 ± 0.17 ^a^	3.63 ± 0.10 ^a^
Total identified	12.31 ± 0.61 ^a^	12.32 ± 0.53 ^a^	12.82 ± 0.53 ^a^	13.31 ± 0.47 ^a^

Statistically significant figures (*p* < 0.05) are indicated with lowercase letters (e.g., a–c) within each row. * n.d.: not detected.

**Table 8:** The eight different parameters of the four extraction techniques to optimize bioactive compounds extraction. Minimum and maximum values are coded with –1 and 1 for the Plackett–Burman design.

Variables	SΤE	PEF	UPAE	UBAE
X1	Solvent choice (0–100%, *C*_EtOH_)	Solvent choice (0–100%, *C*_EtOH_)	Solvent choice (0–100%, *C*_EtOH_)	Solvent choice (0–100%, *C*_EtOH_)
X2	Solvent-to-solid ratio (10–50 mL/g)	Solvent-to-solid ratio (10–50 mL/g)	Solvent-to-solid ratio (10–50 mL/g)	Solvent-to-solid ratio (10–50 mL/g)
X3	Extraction time (30–120 min)	Extraction time (10–30 min)	Extraction time (5–20 min)	Extraction time (5–20 min)
X4	Particle size (0.4–1.2 mm)	Particle size (0.4–1.2 mm)	Particle size (0.4–1.2 mm)	Particle size (0.4–1.2 mm)
X5	Temperature (20–80 °C)	Electric field strength (0.6–1 kV/cm)	Ultrasonic power (60–120 W)	Ultrasonic power (110–220 W)
X6	Stirring speed (250–500 rpm)	Pulse period (100–1000 μs)	Pulsation (12–48 P/min)	Ultrasonic frequency (37–80 kHz)
X7	Magnetic stir bar (10–25 mm)	Pulse duration (10–100 μs)	Probe length position (5–15 mm)	Ultrasonic mode (sweep–pulse)
X8	Number of cycles (1–2 cycles)	Number of cycles (1–2 cycles)	Number of cycles (1–2 cycles)	Number of cycles (1–2 cycles)

## Data Availability

All related data and methods are presented in this paper. Additional inquiries should be addressed to the corresponding author.
